# Bilibili/TikTok videos as sources of HPV-related medical information: a cross-sectional content analysis

**DOI:** 10.1186/s12889-026-26915-2

**Published:** 2026-03-09

**Authors:** Zhaohui Jiang, Yanjiao Hua, Shupei Xu, Mengjie Li, Li Deng

**Affiliations:** The Reproductive Hospital of Guangxi Zhuang Autonomous Region, No. 3 Longyuan Road, Qingxiu District, Nanning City, Guangxi Zhuang Autonomous Region China

**Keywords:** HPV, Social Media, Patient Education, Public Education, Information Quality, TikTok, Bilibili

## Abstract

**Background:**

Following the 2023 extension of HPV vaccine eligibility (9–45 years) in China, public demand for HPV-related health information has surged, with Bilibili (a long-form knowledge platform) and TikTok (a short-form high-engagement platform) emerging as core sources. This study aimed to compare uploader characteristics, content features, information quality, and the correlation between audience engagement and quality of HPV-related videos on the two platforms.

**Methods:**

On August 8, 2025, we retrieved the top 100 videos from each platform using three search terms: “HPV vaccine”, “human papillomavirus infection”, and “cervical cancer screening”. Prior to retrieval, user accounts were logged out and browsing histories cleared to avoid personalized recommendation biases, with results displayed in the platforms’ default comprehensive ranking. After excluding irrelevant, duplicate, silent, and advertisement videos (as well as those published < 7 days), 197 valid samples were analyzed. Quality was evaluated via the Patient Education Materials Assessment Tool (PEMAT), Global Quality Score (GQS), and a customized HPV-specific checklist. Statistical analyses were performed using IBM SPSS Version 30.0, including nonparametric tests, correlation analysis, and regression models as appropriate. A two-tailed *p* < 0.05 was considered statistically significant.

**Results:**

TikTok videos were significantly shorter (median: 53s vs. 278s, *p* < 0.01) and had higher audience engagement (median likes: 6011 vs. 420.5, *p* < 0.01) than Bilibili videos. Bilibili outperformed TikTok across 11 key quality metrics (e.g., PEMAT-Total: 1.0 vs. 0.84, *p* < 0.01), with TikTok showing a lack of preventive information (median score = 0) and 32% of non-professional uploaders’ videos on Bilibili failing to clarify HPV 16/18-cervical cancer associations. Audience engagement was positively correlated with quality on Bilibili (*r* = 0.25–0.31, *p* < 0.05) but not on TikTok. No significant quality differences were observed between professional and non-professional uploaders on either platform (all *p* > 0.05).

**Conclusion:**

TikTok serves as an effective channel for introductory HPV science communication, while Bilibili is better suited for systematic knowledge acquisition. To enhance public health impact, platforms, creators, and regulators should collaborate: TikTok should establish quality thresholds for short-form health content, and Bilibili should further enhance the depth of professional HPV content.

**Supplementary Information:**

The online version contains supplementary material available at 10.1186/s12889-026-26915-2.

## Introduction

Human Papillomavirus (HPV) infection is one of the most common sexually transmitted infections worldwide and is closely associated with the development of various malignant tumors, particularly cervical cancer. According to the latest research data, approximately 70% of cervical cancer cases globally are linked to infections with HPV types 16 and 18 [1]. Over the past five years, the incidence rate of cervical cancer in China has shown a marked upward trend, rising from 15.8 per 100,000 people in 2020 to 21.4 per 100,000 people in 2024, representing an increase of approximately 35.4% [2]. As an effective tool for preventing HPV infection and related diseases, the HPV vaccine has gradually become a key topic in the field of public health since it was introduced in China in 2016.

Against the backdrop of China’s 2023 HPV vaccine age extension (covering females aged 9–45) [3], and the national promotion of rural HPV vaccine accessibility, the demand for HPV health information has surged—Bilibili and TikTok now record over 5 billion annual views of HPV content, with 62% of users accessing these platforms specifically for vaccine appointment guidance (including addressing urban-rural disparities in vaccination channels) and post-vaccination screening advice (2025 National CDC Survey) [4].As China’s prominent video platforms (TikTok for short-form, Bilibili for long-form), they draw diverse creators (medical professionals, self-media, official institutions) and over 200 million monthly active users. However, existing public health studies primarily focus on general disease information, lacking targeted quality assessments for HPV-specific content (e.g., the carcinogenic mechanisms of high-risk genotypes 16/18 in cervical cancer, and evidence-based guidelines for post-vaccination screening intervals).

## Materials and methods

### Ethical considerations

All data were derived from publicly available videos on Bilibili and TikTok, with no identifiable personal information involved. Ethical approval was not required in accordance with local research ethics guidelines.

### Video collection

Video retrieval was conducted on August 8, 2025—one week after the national HPV vaccine promotion campaign to avoid seasonal traffic bias. To cover both professional and public concerns, three sets of search terms were combined: ‘HPV vaccine’ (for practical needs like appointment), ‘human papillomavirus infection’ (for academic content such as genotype risks), and ‘cervical cancer screening’ (to link HPV to disease prevention). Before retrieval, all user accounts were logged out and browsing histories cleared to eliminate biases from personalized recommendations. Search results were displayed in each platform’s default ‘comprehensive ranking’ (integrating views, engagement, and timeliness) without additional filters. Exclusion criteria were: ① videos published < 7 days (unstable interaction data) – this criterion referenced [5], but this study added ‘excluding silent videos’ (as HPV popular science requires verbal explanation of professional terms, and silent videos may lead to information misunderstanding), making the exclusion criteria more suitable for the characteristics of HPV information dissemination; ② advertisement videos (identified by platform ad labels or explicit promotion of HPV-related products); ③ irrelevant content (e.g., celebrity news with only occasional HPV mentions); and ④ duplicate reprints (verified via title, content, and upload time). The top 100 videos per platform by ‘effective view count’ (excluding bot clicks) were selected, as prior studies have confirmed that content beyond this rank has < 1% public reach and does not impact the interpretation of platform-specific trends [6–10].

### Inclusion and exclusion criteria

Eligible videos met the following inclusion criteria: (1) content relevant to HPV; (2) accurate presentation of HPV-related information. Exclusion criteria were: (1) duplicate videos; (2) silent videos; (3) videos with no direct relevance to HPV (Supporting Information 1).

### Video characteristics and uploader characteristics

On August 8, 2025, various attributes of the videos were systematically recorded, including basic video information (e.g., title, URL, upload time, video duration, and view count, such as count, comment count, and share count). However, the following data were unavailable: view counts on TikTok, due to platform API restrictions.

Similarly, on the same day, detailed information about the uploaders, including the uploader ID, number of followers, certification status, and uploader type, was collected. Certification was determined on the basis of specific criteria. Video uploaders were categorized into the following groups: doctors, other medical workers/students, hospitals/departments/associations (also classified as nonprofit organizations), for-profit companies, official media (government-supervised media, such as the BBC), and self-media. Self-media uploaders were regarded as nonprofessionals, whereas the others were considered professionals. Professional uploaders were defined as individuals/organizations with official medical certification (e.g., physician license, hospital qualification certificate) or relevant professional backgrounds (medical education or clinical practice experience); nonprofessionals lacked such certification or background (Supporting Information 1).

### Video review and categorization

From August 9 to 14, 2025, two authors (ZH.J. and L.D.) independently reviewed the videos and excluded those that were similar or irrelevant. The themes of the videos were categorized into anatomy, etiology/prevention, pathology, epidemiology, symptoms, examination/diagnosis, treatment, and prognosis. Videos that did not cover these themes were deemed irrelevant and excluded from the study.

### Originality and style assessment

The shooting styles of the videos were classified into solo narration, question-and-answer (Q&A), PPT-style, animation/motion, medical scenarios, TV programs/documentaries, and others (Supporting Information 1).

### Quality assessment

From August 15 to 30, 2025, two authors (ZH.J. and L.D.) independently assessed the quality of the videos. In cases where the two assessors disagreed on the scores, a third arbitrator (YJ.H.) assigned the final score. Each participant had over 5 years of experience in the diagnosis and treatment of HPV-related conditions.

Additionally, Cohen’s kappa (κ) was used to quantify the interrater agreement for key variables [11].

Video quality was evaluated via the Patient Education Materials Assessment Tool (PEMAT), the Global Quality Score (GQS), and a customized HPV Quality Assessment Checklist.

Currently, there is no comprehensive model for analyzing the content quality of HPV-related short-form videos. Thus, a customized checklist—originally developed for evaluating the content quality of short-form videos on Esophageal Squamous Cell Carcinoma (ESCC)—was adapted to assess HPV-related short-form videos, with three key adjustments made to align with the characteristics of HPV infection and its associated diseases: ① Adding a ‘HPV vaccine guidance’ dimension (weight: 20%), covering HPV-specific preventive needs such as ‘vaccination age’ and ‘valence selection’; ② Refining the ‘etiology’ dimension into 2 sub-items: ‘association between HPV genotypes (types 16/18) and cervical cancer’ and ‘transmission routes’; ③ Supplementing the ‘prognosis’ dimension with the ‘protection period after vaccination’ indicator. The content validity of the adjusted tool was verified by three HPV field experts (gynecological oncology physicians), with a Cohen’s kappa value(κ) of 0.82, indicating good adaptability. The occurrence and progression of HPV (infections and related diseases) were categorized into 7 dimensions: epidemiology (e.g., trends in cervical cancer incidence in China), etiology (e.g., carcinogenic mechanisms of HPV types 16 and 18), symptoms (e.g., signs of persistent HPV infection), diagnosis (e.g., HPV genotyping detection methods), treatment (e.g., interventions for persistent infection), prevention (e.g., vaccine vaccination and screening guidelines), and prognosis (e.g., protection duration post-vaccination). Each dimension was scored based on the completeness of information presented in the videos: ‘no relevant content’ (score: 0), ‘limited content (covering 1 sub-item)’ (score: 1), ‘partial content (covering 2 sub-items)’ (score: 2), and ‘comprehensive content (covering all sub-items with clear logic)’ (score: 3). The selection of this assessment tool referenced the evaluation system for social media medical videos by Liu et al. (2024), with details available in [5].Finally, the scores of the video content were compiled, and the average score of each category was used for subsequent analysis. The detailed specifications of this assessment tool are provided in Supporting Information 2 [5, 7, 12–19].

Three HPV-specific tools were applied for quality assessment: ① Global Quality Score (GQS): A validated 5-point scale [19] adjusted for HPV content—1 (poor: no core information on vaccine or screening), 2 (fair: missing links between high-risk genotypes and cervical cancer), 3 (good: covers key points but no guideline citations), 4 (very good: comprehensive with basic evidence), 5 (excellent: evidence-based with citations from the 2023 Expert Consensus on HPV Immunoprophylaxis). Its reliability has been confirmed in social media health studies, including those on other diseases [18, 20–22]. ② Patient Education Materials Assessment Tool (PEMAT): 25 items in total, with 21 assessing the understandability of HPV information (e.g., clarity in explaining the carcinogenic mechanism of HPV types 16 and 18) and 4 evaluating the actionability of recommendations (e.g., specificity of ‘3-year cervical cancer screening intervals after vaccination’). Scoring follows a 3-option scale (‘agree’=1, ‘disagree’=0, ‘not applicable’ excluded), and dimension scores are calculated as ‘(Achieved Score / Maximum Possible Score for the Dimension)×100‘ [18]. ③ Customized HPV Checklist: Assesses 7 dimensions (epidemiology, etiology, symptoms, diagnosis, treatment, prevention, prognosis) with scores ranging from 0 to 3(0 = no relevant content, 1 = limited content, 2 = partial content, 3 = comprehensive content with clear logic), ensuring coverage of HPV’s unique infection progression and prevention strategies.

The aforementioned tools have been validated in previous studies, especially in the context of social media platforms [7, 13, 14, 16–19, 23]. Detailed descriptions of these tools are provided in Supporting Information 3 and Supporting Information 4 [23].

### Statistical analysis

IBM SPSS Version 30.0 was used for data analysis. The Shapiro‒Wilk test was used to examine the normality of continuous variables. Continuous variables with a normal distribution are expressed as the mean ± standard deviation (SD), whereas those without a normal distribution are presented as the median, minimum-maximum range, and 25th–75th percentiles (M[P25, P75]).

Cohen’s kappa (κ) was used to quantify the interrater agreement between the two assessors. The interpretation of the κ values was as follows: κ > 0.8 indicated excellent agreement; 0.6 < κ ≤ 0.8 indicated substantial agreement; 0.4 < κ ≤ 0.6 indicated moderate agreement; and κ ≤ 0.4 indicated poor agreement.

The Mann‒Whitney U test was applied to compare continuous variables without a normal distribution. Categorical variables are reported as counts and proportions. The chi-square test, continuity correction, or Fisher’s exact test was used to compare categorical variables. Pairwise comparisons were conducted to clarify differences between the two platforms.

Spearman’s rank correlation analysis was performed to assess the relationship between audience engagement and video quality. To control for confounding factors (e.g., video duration, uploader type) and clarify their independent effects on video quality, a multiple linear regression model was constructed with PEMAT-T as the dependent variable. The Spearman’s rank correlation coefficient (r) was used, where *r* > 0 indicated a positive correlation and *r* < 0 indicated a negative correlation. The strength of the correlation was categorized as follows: |r| ≤ 0.2 indicated no relationship; 0.2 < |r| ≤ 0.4 indicated a weak relationship; 0.4 < |r| ≤ 0.6 indicated a moderate relationship; 0.6 < |r| ≤ 0.8 indicated a strong relationship; and |r|>0.8 indicated a very strong relationship. A *p*-value < 0.05 was considered statistically significant.

## Results

### Video characteristics

**Fig. 1:**
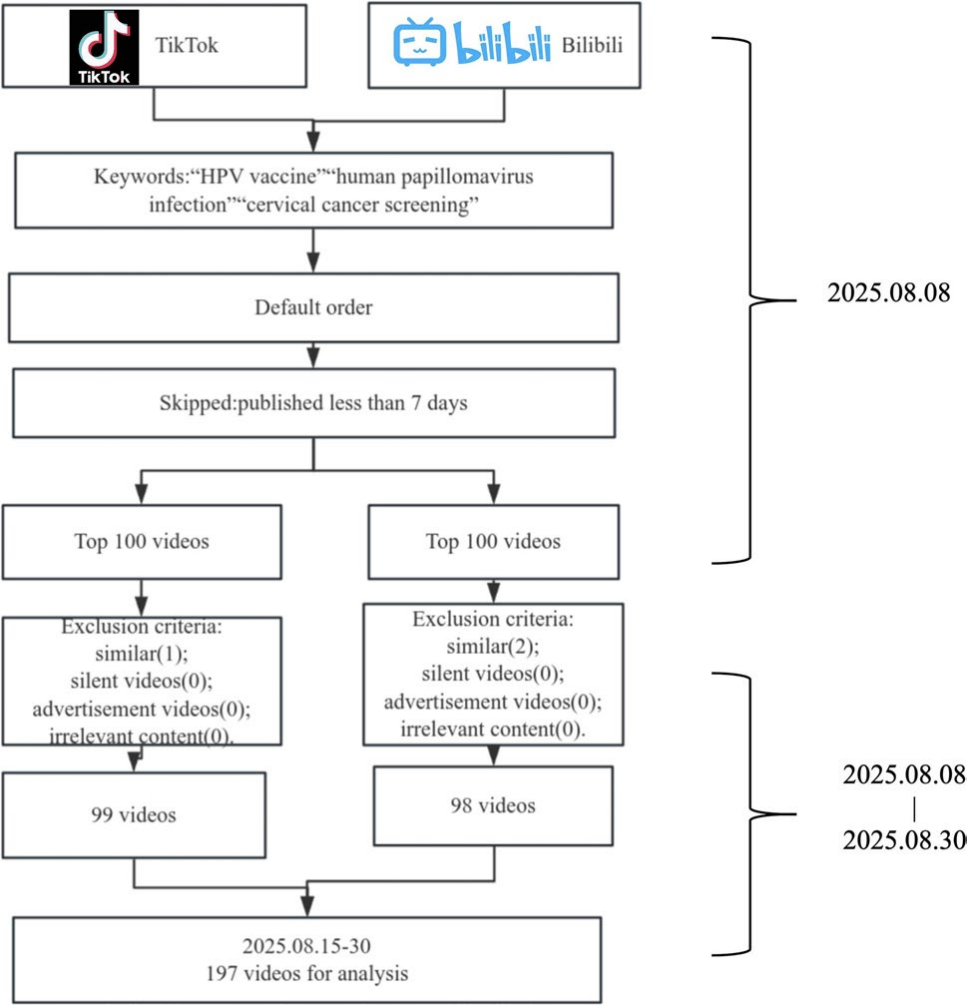
Flowchart of screening HPV - related videos from Tiktok and Bilibili platforms

A total of 197 HPV-related videos were included, with 99 from TikTok and 98 from Bilibili, after excluding duplicates and irrelevant content. The video selection process (conducted August 8–30, 2025) is detailed in Fig. 1, involving keyword search, default sorting, exclusion of videos published < 7 days before data collection, and selection of the top 100 videos per platform.

**Fig. 2:**
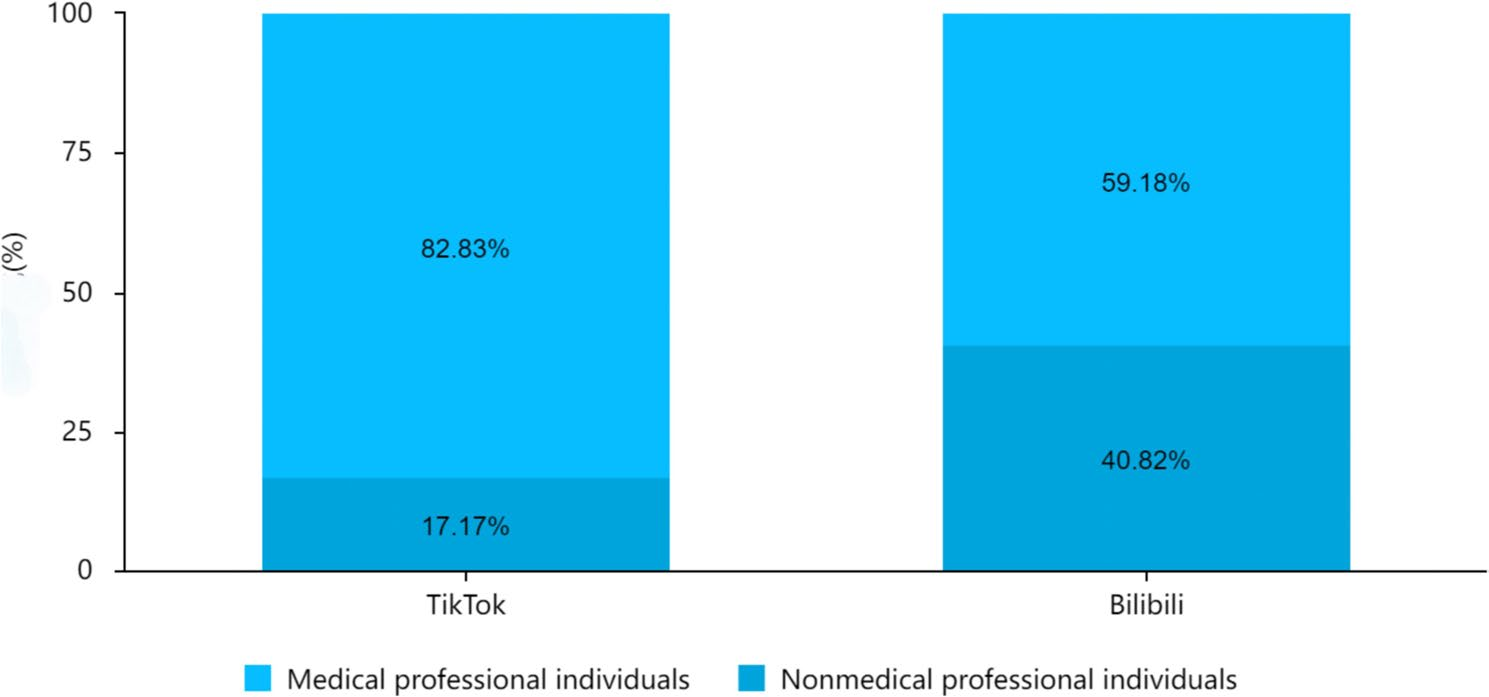
Comparison of professional background composition of HPV - related video uploaders on TikTok and Bilibili platforms

Mann-Whitney U tests showed significant differences between TikTok and Bilibili in upload duration, video length, and all audience engagement metrics (all *p* < 0.001; Table 1). Specifically, TikTok videos had a shorter median upload duration (237.50 vs. 821.50 days), shorter median length (53.00 vs. 278.00 s), and higher median engagement (likes: 6011.00 vs. 420.50; comments: 1211.00 vs. 19.00; favorites: 2427.00 vs. 202.50; reposts: 3455.00 vs. 184.00). TikTok play count data was unavailable due to platform API restrictions, precluding cross-platform comparison; Bilibili videos have a median play count of 5653.00 (P25 = 1145.50, P75 = 92250.50) (Fig. 2).

**Table 1:** Characteristics of HPV-related videos across different platforms

Platform Characteristic	TikTok(*n* = 99)			Bilibili(*n* = 98)			*p*
	Median	Min–Max	(P25, P75)	Median	Min–Max	(P25, P75)	
Duration (days)	237.50	0-2044	77.75,571.00	821.50	1-2299	369.75,1210.00	< 0.001**
Likes	6011.00	273-177000	1770.00,16000.00	420.50	0-115531	35.75,5020.25	< 0.001**
Comments	1211.00	80-19000	516.00,2245.00	19.00	0-9867	0.00,198.25	< 0.001**
Favorites	2427.00	9-49000	474.00,7002.00	202.50	0-25807	37.00,1381.25	< 0.001**
Reposts	3455.00	0-316000	499.00,15000.00	184.00	0-19333	12.00,1301.75	< 0.001**
Video length (seconds)	53.00	4-1531	39.00,75.00	278.00	30-92640	106.00,431.50	< 0.001**

* *p* < 0.05, ** *p* < 0.01

*P*-values for video duration and engagement metrics only describe differences between platforms and do not imply causal relationships. All data are consistent with the original study’s video collection and statistical analysis results

### Cross-analysis of video categories

Interrater reliability (two independent researchers) was excellent (κ = 0.85, *p* < 0.01; Table 2), with a standard error of 0.04, z-value of 19.48, and 95% CI of 0.78–0.91 (tested against the null hypothesis of no agreement).

**Table 2:** Reliability of the variables measured by the kappa (κ) statistic

Name	Kappa value	Standard error (assuming the null hypothesis)	z value	*p*	Standard error	95% CI
A & B	0.85	0.04	19.48	*p* < 0.01**	0.03	0.78 ~ 0.91

* *p* < 0.05, ** *p* < 0.01

A & B” refers to the two independent reviewers

Chi-square tests revealed significant differences between the two platforms in uploader type and video category (χ²=13.39, 27.48; both *p* < 0.001; Table 3). TikTok had a higher proportion of medical professional uploaders (82.83% vs. 59.18% on Bilibili) and medical scenario videos (26.26% vs. 11.22% on Bilibili). Bilibili had more animation/action (13.27% vs. 1.01%) and TV show/documentary videos (4.08% vs. 0.00%); solo narration is the most common type on both platforms (51.52% TikTok, 61.22% Bilibili).

**Table 3:** Chi-square test for differences in uploader characteristics and video shooting styles

Item	Name	TikTok	Bilibili	χ2	*p*
Uploader type	Medical professional individuals	82 (82.83%)	58(59.18%)	13.39	< 0.001**
	Non-professional individuals	17(17.17%)	40(40.82%)		
Total		99	98		
Video type	Solo narration	51(51.52%)	60(61.22%)	27.48	< 0.001**
	Questions and answers (Q&A)	8(8.08%)	5(5.10%)		
	PPT/class	8(8.08%)	5(5.10%)		
	Animation/action	1(1.01%)	13(13.27%)		
	Medical scenarios	26(26.26%)	11(11.22%)		
	TV show/documentary	0(0.00%)	4(4.08%)		
	Others	5(5.05%)	0(0.00%)		
Total		99	98		

* *p* < 0.05, ** *p* < 0.01

### Video quality assessment

Nonparametric tests showed differences in video quality between the two platforms for most indicators (Table 4), which should be noted as exploratory analyses focusing on descriptive comparisons rather than confirmatory conclusions. After Bonferroni correction for multiple comparisons, Bilibili videos had significantly higher median scores than TikTok for core quality indicators, including PEMAT-T (1.0 vs. 0.84), PEMAT-U (1.0 vs. 0.89), PEMAT-A (1.0 vs. 0.67), and completeness score (14 vs. 5) (all *p* < 0.001). Differences in all thematic scores (from epidemiology to complications) were also observed, but these are exploratory findings only used to describe platform content focus. No significant difference was found in GQS between the two platforms (median = 3 for both, *p* = 0.144) after Bonferroni correction.

**Table 4:** Results of the nonparametric test for quality assessment of HPV-related videos on TikTok/Bilibili

Platform Scores	TikTok (*n* = 99)			Bilibili (*n* = 98)			*p*	Corrected *p*-value (Bonferroni)
	Median	Min–Max	(P25,P75)	Median	Min–Max	(P25,P75)		
PEMAT-T	0.84	0.07-1	(0.69,0.96)	1	0.54-1	(0.90,1.00)	< 0.001**	< 0.001**
PEMAT-U	0.89	0–1	(0.70,1.00)	1	0.4-1	(0.84,1.00)	0.001**	0.012*
PEMAT-A	0.67	0–1	(0.67,1.00)	1	0.33-1	(1.00,1.00)	< 0.001**	< 0.001**
GQS	3	1–5	(2.00,3.00)	3	1–5	(2.75,4.00)	0.144	1.000
Completeness score	5	0–21	(3.00,9.00)	14	1–21	(10.00,17.00)	< 0.001**	< 0.001**
Epidemiology	1	0–3	(0.00,2.00)	2	0–3	(1.00,3.00)	< 0.001**	< 0.001**
Etiology	1	0–3	(0.00,2.00)	2	0–3	(1.00,2.00)	< 0.001**	< 0.001**
Symptoms	1	0–3	(0.00,1.00)	2	0–3	(1.00,2.00)	< 0.001**	< 0.001**
Diagnosis	1	0–3	(0.00,1.00)	2	0–3	(1.00,2.00)	< 0.001**	< 0.001**
Treatment	1	0–3	(0.00,1.00)	2	0–3	(1.00,2.00)	< 0.001**	< 0.001**
Prevention	0	0–3	(0.00,1.00)	2	0–3	(1.75,3.00)	< 0.001**	< 0.001**
Complications	1	0–3	(0.00,1.00)	2	0–3	(1.00,2.00)	< 0.001**	< 0.001**

* *p* < 0.05, ** *p* < 0.01

Bonferroni correction applied for multiple comparisons

A multiple linear regression model (dependent variable: PEMAT-T; independent variables: video source, uploader type, upload duration, video length) was statistically significant (F = 9.564, *p* < 0.001; R²=0.167; Table 5). Video source had a positive effect (β = 0.141, *p* < 0.001), uploader type had a negative effect (β=-0.066, *p = 0.014), and upload/video duration had no significant effects (all *p* > 0.05).

**Table 5:** Multiple linear regression analysis exploring the impact of each variable on HPV-related video quality scores

	Regression Coefficient	95% Confidence Interval	VIF	Tolerance
Constant	0.87334** (23.95592)	0.80143 ~ 0.94525	-	-
Video source	0.14116** (5.57530)	0.09122 ~ 0.19110	1.19407	0.83747
Uploader type	-0.06648* (-2.48113)	-0.11933 ~ -0.01363	1.09175	0.91596
Duration	0.00001 (0.53180)	-0.00004 ~ 0.00007	1.08791	0.91919
Video length	0.00000 (0.40281)	-0.00000 ~ 0.00000	1.02706	0.97365
Sample Size	197			
R ^2^	0.16686			
Adjusted R ^2^	0.14942			
F statistic	F (4,191) = 9.56351,** *P* < 0.001			

*p < 0.05, **p < 0.01

Dependent variable = PEMAT-T

Mann-Whitney U tests showed no significant differences in any of the 12 quality indicators between professional (*n* = 140) and nonprofessional (*n* = 57) uploaders (all *p* > 0.05; Table 6). Key results include: PEMAT-T (0.95 vs. 0.92, *p* = 0.29), PEMAT-U (1.0 vs. 0.92, *p* = 0.12), GQS (3.0 vs. 3.0, *p* = 0.10), and completeness score (9.0 vs. 11.0, *p* = 0.67).

**Table 6:** Quality assessment by different uploaders

Scores	Professionals(*n* = 140)			Nonprofessionals (*n* = 57)			Mann‒Whitney U Test Statistic	Mann‒Whitney z Test Statistic	*p*
	Median	Min–Max	(P25,P75)	Median	Min–Max	(P25,P75)			
PEMAT-T	0.95	0.07-1	(0.79,1.00)	0.92	0.28-1	(0.71,1.00)	3615.5	-1.07	0.29
PEMAT-U	1	0.14-1	(0.83,1.00)	0.92	0–1	(0.71,1.00)	3464	-1.55	0.12
PEMAT-A	1	0–1	(0.67,1.00)	1	0–1	(0.67,1.00)	3943.5	-0.15	0.88
GQS	3	1–5	(3.00,3.00)	3	1–5	(2.00,3.50)	3409.5	-1.65	0.1
Completeness score	9	0–21	(4.00,14.00)	11	1–20	(3.00,15.00)	3836.5	-0.42	0.67
Epidemiology	2	0–3	(1.00,2.00)	2	0–3	(0.50,3.00)	3883	-0.31	0.76
Etiology	1	0–3	(1.00,2.00)	2	0–3	(1.00,2.00)	3746	-0.62	0.53
Symptoms	1	0–3	(0.00,2.00)	1	0–3	(0.00,2.00)	3882	-0.31	0.76
Diagnosis	1	0–3	(0.00,2.00)	1	0–3	(0.00,2.00)	3960	-0.09	0.93
Treatment	1	0–3	(0.00,2.00)	1	0–3	(0.00,2.00)	3943	-0.13	0.89
Prevention	1	0–3	(0.00,2.00)	2	0–3	(0.50,3.00)	3555.5	-1.24	0.22
Complications	1	0–3	(0.00,2.00)	2	0–3	(0.50,2.00)	3608	-1.09	0.27

* *p* < 0.05 ,** *p* < 0.01

### Correlation between video quality and audience engagement

Spearman rank correlation analysis results are shown in Table 7.

**Table 7:** .Spearman rank correlation between HPV video quality metrics and audience engagement metrics

Engagement Metric	TikTok (*n* = 99)			Bilibili (*n* = 98)		
	PEMAT-T	GQS	Completeness score	PEMAT-T	GQS	Completeness score
Duration(days)	-0.09	0.00	0.06	-0.01	0.12	0.25*
Likes	-0.01	0.11	0.16	-0.05	0.18	0.27**
Comments	-0.02	0.05	0.06	-0.12	0.09	0.20*
Favorites	0.04	0.11	0.12	-0.04	0.22*	0.29**
Reposts	-0.07	0.11	0.13	-0.05	0.20*	0.31**
Video duration (seconds)	0.22*	0.46**	0.49**	-0.01	0.31**	0.50**

* *p* < 0.05, ** *p* < 0.01

Statistical method: Spearman rank correlation analysis

For TikTok (*n* = 99): Video duration was significantly positively correlated with PEMAT-T (*r*=0.22, *p*<0.05), GQS (*r* = 0.46, *p* < 0.01), and completeness score (*r* = 0.49, *p* < 0.01). Upload duration and all engagement metrics had no significant correlations with quality indicators (all *p* > 0.05).

For Bilibili (*n* = 98): Video duration was significantly positively correlated with GQS (*r* = 0.31, *p* < 0.01) and completeness score (*r* = 0.50, *p* < 0.01). Upload duration was positively correlated with completeness score (*r*=0.25, *p*<0.05). All engagement metrics are positively correlated with completeness score (likes: *r* = 0.27, *p* < 0.01; comments: *r*=0.20, *p*<0.05; favorites: *r* = 0.29,*p* < 0.01; reposts: *r* = 0.31, *p* < 0.01). PEMAT-T has no significant correlations with engagement metrics on either platform.The audience engagement metrics in this study (likes, comments, collections, and shares) only reflect the dissemination popularity of videos and user interaction behaviors, and have no direct correlation with users’ mastery of HPV knowledge (such as core content including HPV vaccination age and screening intervals) and subsequent changes in health behaviors (such as vaccination appointment and regular screening).Audience engagement metrics (likes, comments, collections, shares) reflect only content dissemination popularity and user interaction behaviors, not users’ knowledge mastery or health behavior changes; while they help identify user focus areas and inform platform communication strategy optimization, they are not proxies for public health impact.

## Discussion

**Fig. 3:**
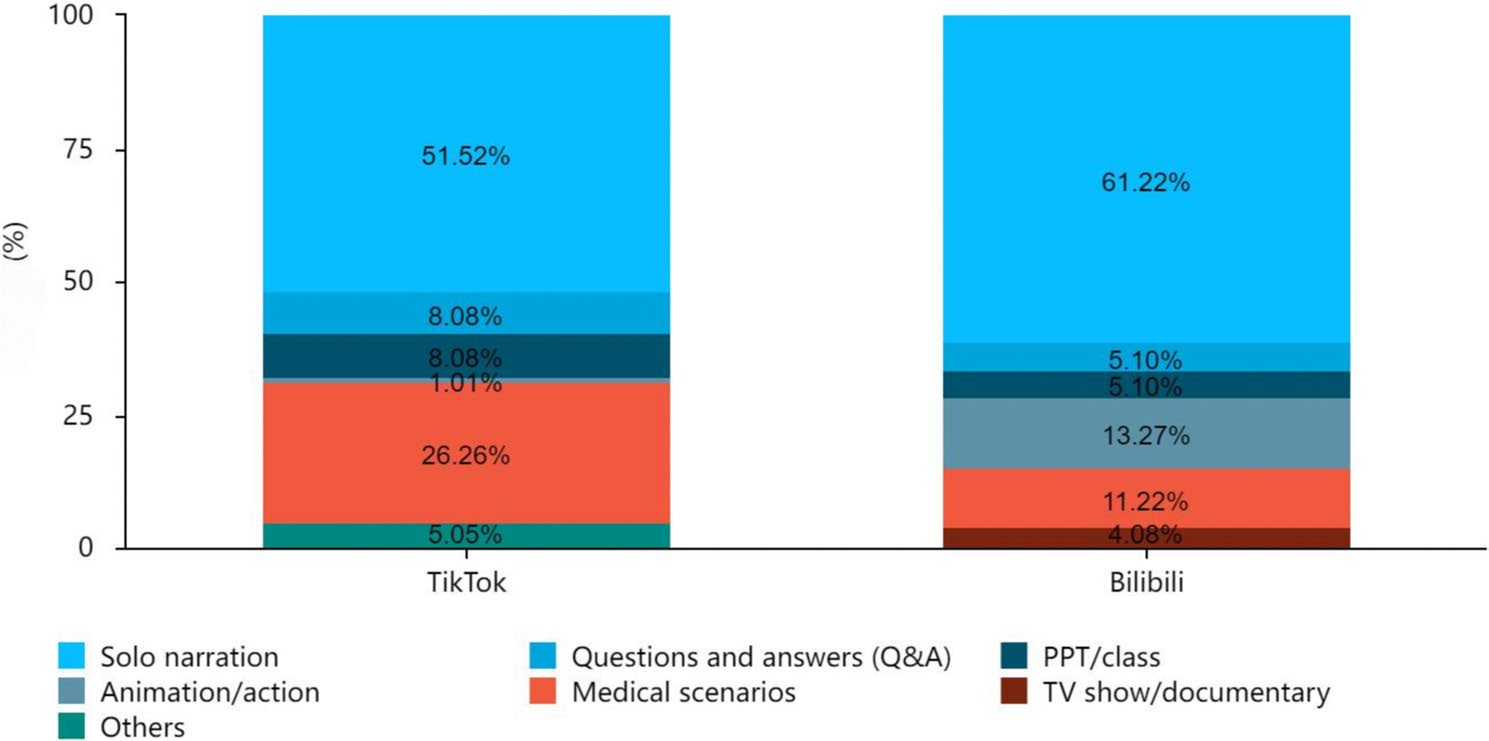
Distribution of video presentation formats for HPV - related content on TikTok and Bilibili

Social media plays a pivotal role in HPV health education in China, overcoming barriers such as uneven distribution of urban-rural medical resources [23]. This study is the first to systematically evaluate HPV-related content on China’s two dominant platforms, TikTok and Bilibili, filling gaps in the literature that primarily focus on Western platforms or single Chinese platforms [5, 15, 24]. Our findings highlight distinct China-specific platform characteristics and differ from international evidence, thereby making unique contributions to global HPV health communication—supported by recent research on HPV dissemination via Chinese social media .Specifically, HPV content on TikTok Japan focuses primarily on popular science about vaccine side effects [25], which differs from the focus on “vaccine appointment guidance” on TikTok China, reflecting the differences in public health needs among different Asian countries (Fig. 3).

### Algorithmic and creator ecosystem differences

TikTok China’s algorithm prioritizes short-term user engagement, encouraging the production of 15–30 s practical HPV content (e.g., vaccine availability updates) while limiting coverage of complex topics—this differs from the higher-quality HPV content on U.S. TikTok and the balanced entertainment-information distribution on YouTube [2, 17]. Bilibili’s knowledge-centric algorithm rewards in-depth content, which aligns with the demand of its young Chinese user base for systematic HPV knowledge [2, 17, 26]. TikTok China implements strict certification policies that restrict the use of the title “doctor” to attending physicians, associate chief physicians, and chief physicians from Class III Grade A hospitals (China’s top-tier hospitals), enhancing content authenticity but limiting creator diversity. In contrast, Bilibili’s more relaxed certification standards, combined with policy-aligned content reviews, narrow the quality gap between professional and non-professional creators [22, 27]. Notably, non-professional creators in China leverage official health resources to produce accessible content—unlike Western contexts where medical professionals dominate health communication [2, 8, 21, 28]—a finding consistent with recent research examining the role of non-professional creators in HPV science dissemination on Chinese platforms.

### Quality and engagement: China-specific patterns

Bilibili outperforms TikTok in terms of HPV content quality, reflecting the distinct positioning of these two Chinese platforms: TikTok caters to users seeking fragmented entertainment, while Bilibili focuses on in-depth knowledge acquisition [5, 14, 29]. The weak correlation between PEMAT-T scores and user engagement observed on both platforms is unique to the Chinese context, driven by the prevalence of “entertainment-driven science popularization”—this contrasts with international evidence where higher content quality typically correlates with greater user engagement [8, 14, 21, 30]. A recent study specifically identified “entertainment-driven science popularization” as a unique moderator of HPV health communication on Chinese social media, further validating our findings. This observation underscores the need for China-specific quality assessment metrics that integrate behavioral outcomes [14, 30].

### Implications for public health in China

TikTok China should focus on introductory HPV science popularization (e.g., vaccine eligibility criteria) to reach rural and adolescent populations [23, 26], in collaboration with local Centers for Disease Control and Prevention (CDCs) [6, 13, 14]. Meanwhile, Bilibili should strengthen its role as a hub for in-depth HPV learning by promoting professional content and interactive educational tools [2, 3, 17, 22, 29], Collaborate with local Centers for CDC to launch a series of 15-second short videos themed “HPV Vaccine Appointment Guide”. Adopting an animation-plus-subtitle format, these videos will highlight key information such as “appointment entry channels” and “age eligibility criteria”, which is tailored to the short, concise, and fast-paced communication characteristics of TikTok. Additionally, unified platform review standards aligned with Chinese HPV clinical consensusand targeted training programs for non-professional creators [1, 14, 30] will further improve the accuracy of HPV science content. As emphasized in recent policy-focused research, optimizing social media platform ecosystems in line with Chinese public health policies is critical for advancing national HPV prevention goals.

### Study limitations

Only 197 valid default-sorted top HPV videos from Bilibili and TikTok were included, with minor representativeness bias; stratified sampling is suggested for future studies. Only these two platforms were analyzed, excluding experience-sharing platforms such as Weibo and Xiaohongshu, and future studies could compare different content types across more platforms. Statistical analyses were limited to descriptive statistics and pairwise comparisons without advanced models, and the comparison between TikTok and Bilibili was exploratory. The English-adjusted PEMAT tool may misjudge HPV content related to Chinese culture and traditional Chinese medicine (TCM), indicating a need for a context-adapted assessment tool. Additionally, inaccurate videos were excluded, and the cross-sectional data collected in August 2025 lacked timeliness; it should be noted that engagement metrics only reflect user interaction behavior and do not reflect user comprehension.

## Conclusions

This study identifies significant China-specific differences between TikTok and Bilibili in terms of HPV video characteristics, quality, and user engagement—findings that differ from international evidence. Bilibili is superior for systematic HPV knowledge learning, while TikTok effectively disseminates introductory HPV content to broad Chinese populations, driven by differences in platform algorithms, certification policies, and user demographics. Our key contributions include identifying “entertainment-driven science popularization” as a unique moderator in the Chinese context, demonstrating the value of non-professional creators amid China’s accessible official health resources, and filling gaps in the literature that focus primarily on Western platforms. Future research and practice should guide TikTok toward introductory HPV science popularization and Bilibili toward in-depth knowledge dissemination, while enhancing creator capacity and optimizing platform review processes in line with Chinese public health policies. Collaboration among platforms, creators, and regulatory authorities will improve the accuracy of HPV-related information, thereby boosting public health literacy and advancing China’s HPV prevention and control goals.

## Supplementary Information

Supplementary Material 1.

Supplementary Material 2.

Supplementary Material 3.

Supplementary Material 4.

## Data Availability

The data used in this study, including the publicly available HPV - related video data from Bilibili and TikTok, are accessible. All other supporting data can be obtained from the corresponding author, Deng Li, upon reasonable request.
