# Supplementary material for: Bilibili/TikTok videos as sources of HPV-related medical information: a cross-sectional content analysis
Source: BMC Public Health. 2026 Mar 9;26:1225. doi: 10.1186/s12889-026-26915-2 (PMC13081635; doi:10.1186/s12889-026-26915-2)
Supplement: Supplementary file 1 — Supplementary Material 1. [file 12889_2026_26915_MOESM1_ESM.doc]

**Details in Methods**

**1. Certification**

Meeting any one of the following conditions can be regarded as certification.

**1.1 TikTok**

①Yellow V: personal certification for a famous person/popular account/ occupation.

On TikTok, only attending/associate/chief doctors who work in a grade 3 and first-class hospital (a hospital ranking system in China, grade 3 and first-class means the top level) can apply for a grey V. Resident doctors or any doctors who do not work in the grade 3 and first-class hospital can **NOT** apply it, and are NOT allowed to upload health-related videos on TikTok.

This strict rule of certification for doctors came into effect in June.2023.

For example:

There is a yellow V on the right of its ID. This is a chief doctor with 1425000 followers.


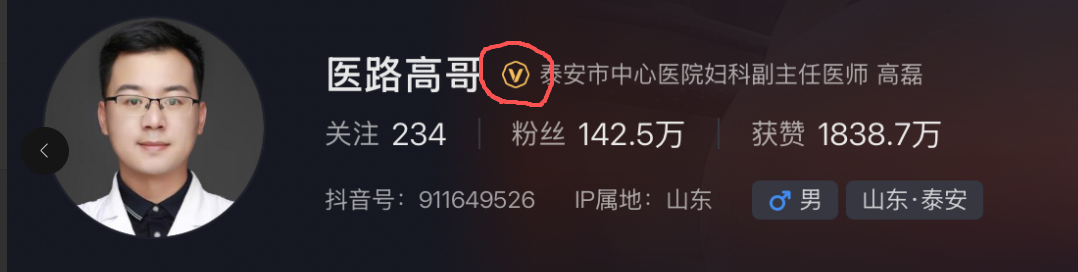


② Blue V: certification for groups.

For example:

There is a blue V on the right of its ID. This is an account from an official media, CCTV, with now 3072 thousand followers.


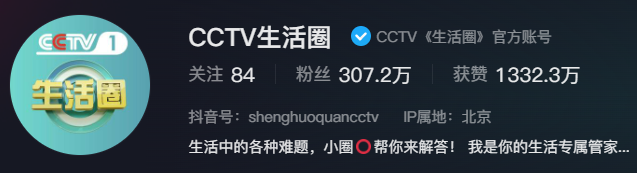


**1.2 Bilibili**

① Yellow flash: personal certification for a famous person/popular account.

For example:


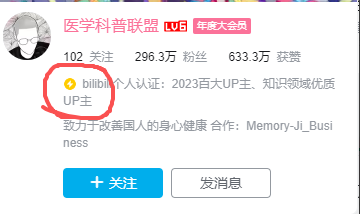
There is a yellow flash on the lower right corner of its icon. This is a popular personal account on Bilibili with 296.3 thousand followers.

② Blue flash: certification for groups.

For example:

There is a blue flash on the right corner of its icon. This is an account from Official Account of [CCTV.com](https://cctv.com/) News Channel with 213.3 thousand followers.


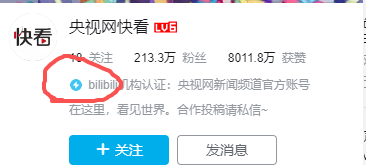


③ Grey V: personal occupational certification such as a doctor.Example：We can not see any yellow or blue flash on his icon. Then, we click on his homepage. We can see a grey V on the right of his homepage, which shows that he is a doctor. (doctor in Chinese: “医生” or “医师”)On Bilibili, individuals who hold both a medical practitioner certificate and an employment certificate from any hospital (for those involved in HPV-related diagnosis and treatment, additional clarification on HPV-related professional scopes is required, e.g., “departments related to HPV such as dermatology and gynecology”) are eligible to apply for a grey V..


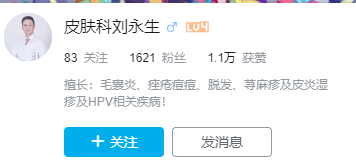

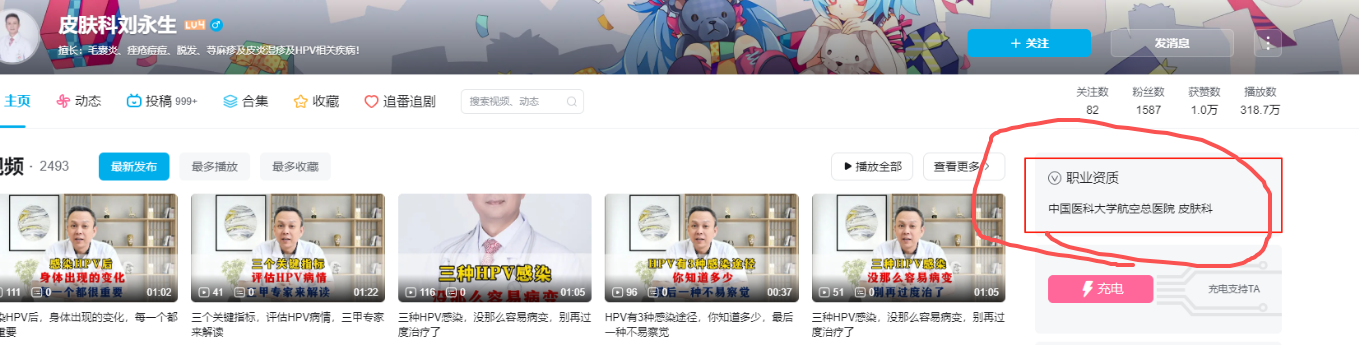


④ In theory, one account may get both the yellow flash and the grey V, but we could not find such an account in our study. On Bilibili, a doctor can apply for a grey V, but only when he gets enough followers (more than 50~100 thousand) can he apply for a yellow flash.

**2. Inclusion and exclusion criteria**

**2.1 Inclusion criteria**

① Searching Date: August 8, 2025

② Searching keywords: Combined keywords “HPV vaccine”, “human papillomavirus infection”, and “cervical cancer screening” (covering HPV-related practical needs, academic content, and disease prevention associations to avoid missing key content with a single keyword)③ Order: the default order without any filtering criteria④ No “sponsored” label (see 2.1).

⑤ Published over a week. (According to the services from the platforms, the data on views and likes are not stable during the first week and can not accurately reflect audience engagement.)

After the inclusion criteria, we had the top 100 videos from each platform (2×100 = 200 videos). However, we had not watched the whole video yet and did not know whether a video was duplicated or irrelevant. Then, we moved to the exclusion criteria part.

After the exclusion criteria, there were 197 videos left (see Figure 1).

**2.2 Exclusion criteria**

① Similar videos. If two videos are similar (completely the same or edited from the same resource), the one uploaded by a certified account remains. If neither video was certified, the one uploaded first remains. Criteria for determining duplication: Verified by consistency in video title, core content (e.g., coverage of HPV knowledge points), and upload time to avoid misjudging duplication based solely on similar titles② Irrelevant videos. The topic of the videos was categorized as anatomy, etiology/ prevention, pathology, epidemiology, symptoms, examinations/ diagnosis, treatment, and prognosis. Videos not covering any of these topics were deemed irrelevant.

**3. Style of video shooting**

① Solo narration: Only one person speaks in the video without other scenarios. Example：https://www.bilibili.com/video/BV1pd4y127Ei/?spm_id_from=333.337.search-card.all.click&vd_source=cc4eded49b3de8468830832f7a750499

②Questions and answers (Q&A): One person asks questions (or questions written in subtitles), and the other answers. Example: https://www.bilibili.com/video/BV15m421j7AP/?spm_id_from=333.337.search-card.all.click&vd_source=cc4eded49b3de8468830832f7a750499

③PPT/class: The videomaker uses PPT to show the video, such as some online medical classes for medical students. Example: https://www.bilibili.com/video/BV1vX53z2EyP/?spm_id_from=333.337.search-card.all.click

④ Animation/action: Examples: https://www.bilibili.com/video/BV1NnKkzhEXW/?spm_id_from=333.337.search-card.all.click&vd_source=cc4eded49b3de8468830832f7a750499

⑤ Medical scenarios: The scenarios include but are not limited to when a patient is receiving treatment or consulting a doctor. Examples: https://www.bilibili.com/video/BV17V4y1T7AX/?spm_id_from=333.337.search-card.all.click&vd_source=cc4eded49b3de8468830832f7a750499

⑥ TV show/documentary: Example:https://www.bilibili.com/video/BV17R4y1A785/?spm_id_from=333.337.search-card.all.click&vd_source=cc4eded49b3de8468830832f7a750499
