# Supplementary material for: Bilibili/TikTok videos as sources of HPV-related medical information: a cross-sectional content analysis
Source: BMC Public Health. 2026 Mar 9;26:1225. doi: 10.1186/s12889-026-26915-2 (PMC13081635; doi:10.1186/s12889-026-26915-2)
Supplement: Supplementary file 2 — Supplementary Material 2. [file 12889_2026_26915_MOESM2_ESM.doc]

**Details in Assessment Tools**

**1.HPV Short-video Content Scorecard**

Currently, there is no comprehensive model to analyze the content quality of HPV short videos. We customized a checklist with reference to Liu et al. (2024)’s social media medical video evaluation framework [1] and relevant literature on Esophageal Squamous Cell Carcinoma (ESCC) short video assessment [2-7] to assess the content quality of HPV short videos.. We divided the occurrence process of HPV into 7 categories, including Epidemiology, Etiology, Symptoms, Diagnosis, Treatment, Prevention, and Outcomes. Then, we score each part of the short video based on the completeness of the content mentioned in the short video. The description of the short video content is classified into No content, Partial content (covering 1 item), Substantial content (covering 2 items), and Comprehensive content (covering all items with clear logic), with a scoring range from 0 to 3 points. Finally, we calculate the score of the video content and use the average score of each part in each group for analysis.More details shown in the Excel (Additional file 3).

**2. GQS** Global Quality Score

The total score is 1 to 5.

【Score 1】Poor quality, poor flow of the site, most information missing, not at all useful for patients.

【Score 2】Generally poor quality and poor flow, some information listed but many important topics missing, of very limited use to patients

【Score 3】Moderate quality, suboptimal flow, some important information is adequately discussed but others poorly discussed, somewhat useful for patients

【Score 4】Good quality and generally good flow, most of the relevant information is listed, but some topics not covered, useful for patients

【Score 5】Excellent quality and excellent flow, very useful for patients

Bernard invented the GQS tool in 2007 to rate the overall quality of a video[7]. Apart from overall quality, it takes into account the flow. Of all the tools, this is the simplest. Since then, the GQS tool have been widely used (see the screenshot of the Pubmed searching page below, the most used tool).


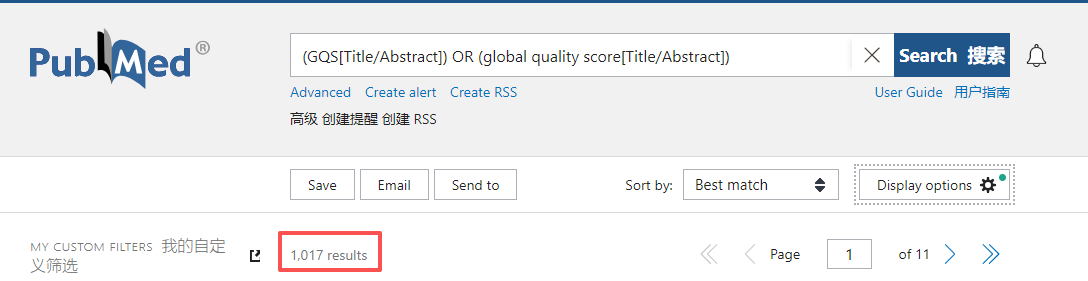


Advantage: ① Quick. ② Widest use.

Disadvantage: ① Limited questions. ② Not quantified clearly.

**3. PEMAT** Patient education materials assessment tool

Because of the complexity of this tool, it can not be shown in the Word, more details shown in the Excel (Additional file 4, or http://www.ahrq.gov/pemat ).

The PEMAT tool is the most comprehensive tool among the four tools in our study. Shoemaker created this tool in 2014 and confirmed its strong internal consistency, reliability, and evidence of construct validity[8]. Further study also confirmed its moderate to excellent interrater reliability[9]. Since then, the PEMAT tool has been widely used (see the screenshot of the Pubmed search page below).


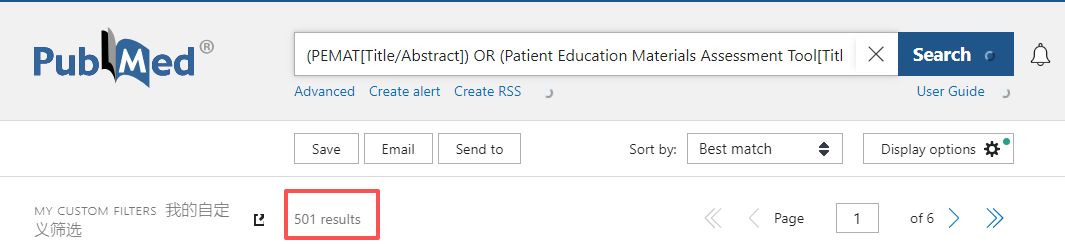


Advantage: ① Most detailed tool. ② Wide use. ③ Reliability and validity confirmed.

Disadvantage: ① Complex and time-consuming. ② “NA (non-applicable)” items potentially resulting in inflating score.

**References:**

1. Liu Z, Chen Y, Lin Y, et al. YouTube/ Bilibili/ TikTok videos as sources of medical information on laryngeal carcinoma: cross-sectional content analysis study. BMC Public Health. 2024;24(1):1594.

2. World Health Organization. Human papillomavirus (HPV) vaccination: WHO position paper, 2020 [Online]. [https://www.who.int/publications/i/item/WHO-ESP-PHP-01.1](https://www.who.int/publications/i/item/WHO-ESP-PHP-01.1).

3. National Health Commission of the People's Republic of China. Comprehensive Prevention and Control Guidelines for Cervical Cancer (2023 Edition). Beijing: People's Medical Publishing House, 2023.

4. National Health Commission of the People's Republic of China. Notice on Expanding the Age Range of HPV Vaccine Vaccination (2023). Beijing: National Health Commission, 2023.

5. Chinese Society of Obstetrics and Gynecology, Chinese Medical Association. Expert Consensus on HPV Immunoprophylaxis (2023). Chin J Obstet Gynecol, 2023, 58(6): 401-408.3.

6. Chinese Society of Obstetrics and Gynecology, Chinese Medical Association. Interpretation of guidelines for cervical cancer screening and management of abnormalities. Chin J Pract Gynecol Obstet, 2022, 38(10): 1021-1026.

7. Centers for Disease Control and Prevention (CDC). HPV Vaccination Recommendations and Guidelines [Online]. [https://www.cdc.gov/hpv/parents/vaccine.html](https://www.cdc.gov/hpv/parents/vaccine.html).

8. Shoemaker SJ, Wolf MS, Brach C. Development of the Patient Education Materials Assessment Tool (PEMAT): a new measure of understandability and actionability for print and audiovisual patient information. Patient Educ Couns. 2014; 96(3):395-403.

9. Vishnevetsky J, Walters CB, Tan KS. Interrater reliability of the Patient Education Materials Assessment Tool (PEMAT). Patient Educ Couns. 2018; 101(3):490-496.
